# Exploring the relationship between space weather conditions and power performance of EgyptSat-1 using machine learning

**DOI:** 10.1038/s41598-025-29518-w

**Published:** 2025-12-12

**Authors:** Dalia Elfiky, Mohammed Abu Bakr Ali, Marwa S. Mostafa, N. Hesham, Ayman Mahmoud Ahmed, Sara K. Ibrahim

**Affiliations:** 1 National Authority for Remote Sensing and Space Science (NARSS), 23 Jozif Tito St, Cairo, 11769 Egypt; 2 Egyptian Space Agency (EgSA), Cairo, 1564 Egypt; 3 Department of Computer & Systems Engineering, Faculty of Engineering, Zagazig University, Zagazig, 44519 Egypt

**Keywords:** EgyptSat-1 power performance, Space weather parameters, 1D- convolutional neural networks (CNN), Long Short-Term memory (LSTM) networks, Random forest regressor, And an ensemble model, Engineering, Mathematics and computing

## Abstract

The growing reliance on satellites highlights the need to understand how space weather quantitatively impacts the reliability and efficiency of power subsystems. While it is well established that space weather disturbances can trigger anomalies in satellite operations, most existing studies lack integrated, data-driven approaches capable of capturing the complex, nonlinear interactions between space weather parameters and satellite health telemetry. This study addresses this gap by introducing a novel four-stage data driven workflow to examine the relationship between key space weather indicators (proton flux, AL index, galactic cosmic rays (GCR), and solar wind density) and the NN1_Voltage and TBS1_Current and temperature of the EgyptSat-1 satellite power subsystem (T1BS, T3BS). The workflow includes: (1) data preprocessing; (2) To handle the high dimensionality and complexity of the data, a two-stage non-linear feature selection approach was employed. In the first stage, an unsupervised Restricted Boltzmann Machine (RBM) was applied to extract a compact and structurally stable feature subset. This was followed by a supervised mutual information (MI) validation step to ensure maximum predictive relevance to the satellite target parameters (T1BS and T3BS). (3) six machine learning models namely: Convolutional Neural Networks (CNN), Long Short-Term Memory (LSTM) networks, Random Forest Regressor, Adaptive Boost, Gradient Boosting, and Voting Regressor, to capture dynamic system behaviours; and (4) anomaly detection and validation by correlating prediction residuals with space weather disturbances, using STL decomposition and Z-score for GCR and P10 anomaly detection, and coincidence rate analysis to assess temporal alignment. The Random Forest (RF) model exhibited strong predictive performance. For NN1_Voltage, the mean squared error (MSE) was 0.00147 (95% CI: 0.00120–0.00184), the root mean squared error (RMSE) was 0.038 (95% CI: 0.0346–0.0428), the mean absolute error (MAE) was 0.028 (95% CI: 0.026–0.031), and the mean absolute percentage error (MAPE) was 0.09% (95% CI: 0.08–0.10%). For TBS1_Current, RF achieved an MSE of 0.0405 (95% CI: 0.0319–0.0489), RMSE of 0.201 (95% CI: 0.179–0.221), MAE of 0.153 (95% CI: 0.136–0.169), and MAPE of 2.4% (95% CI: 2.1–2.6%). Furthermore, analysis of detected anomalies revealed temporal coincidence rates of 31% with GCR disturbances and 27% with P10 proton events. Statistical validation using chi-squared and Fisher’s exact tests yielded significant p-values (e.g., *p* = 2.83 × 10⁻³ for GCR; *p* = 5.63 × 10⁻⁷ for P10), suggesting a potential relationship worth further investigation. This analysis is particularly relevant for assessing unexplained satellite failures such as the loss of EgyptSat-1 and contributes to improved resilience and monitoring strategies for future missions. While the proposed workflow shows strong predictive performance, its validation is currently limited to a single satellite dataset, highlighting the need for broader cross-mission testing. This study not only enhances our understanding of space weather impacts on satellite power systems but also demonstrates the potential of machine learning in improving anomaly detection and resilience of satellites operating in challenging space environments.

## Introduction

The intricate structure of spacecrafts, coupled with the dynamic space environment encompassing phenomena such as solar flares, geomagnetic storms, cosmic ray influxes, and the persistent risk of collisions with space debris, poses a significant challenge for satellite operations and functionality^1,2^. Enhancing the reliability of space system components alone is insufficient to eliminate the possibility of failure. Moreover, the considerable distance between the spacecraft and ground stations often renders the inspection and repair of damaged components unfeasible^3^. This reality underscores the need for effective data monitoring strategies to maintain system functionality and promptly detect and diagnose anomalies. Telemetry data comprises complex, multivariate, heterogeneous, and multi-modal time series, representing the dynamic states of spacecraft subsystems^4^. This study focuses on EgyptSat-1’s power subsystem due to its unexplained loss and the availability of telemetry data, aiming to explore the potential influence of space weather on performance degradation.

Space weather remains a major source of risk for satellite systems. Phenomena such as geomagnetic storms, solar energetic particles (SEP), and galactic cosmic rays (GCR) can collectively induce voltage instabilities^1^, degrade solar panels through displacement damage dose (DDD) effects, and disrupt power regulation processes^5^. These disturbances often lead to anomalies that compromise performance and, in severe cases, trigger partial or complete mission failure^6^.

Satellite monitoring enables early detection of anomalies to prevent failures. Telemetry anomalies, caused by space weather, system faults, or sensor errors, require precise diagnosis. Anomalies, defined as deviations from expected behaviour^7^, are categorized as point anomalies (individual deviations), contextual anomalies (abnormal under specific conditions, e.g., high solar radiation in an eclipse), and collective anomalies (groups of instances abnormal together but not individually). Given the sequential nature of telemetry data, time-series methods are well-suited for anomaly detection. The dynamic, nonlinear characteristics of satellite telemetry have motivated the use of machine learning (ML) models, which have shown strong associations between geomagnetic indices such as Dst, Kp, and Ap and satellite anomalies^8^, underscoring the need for data-driven approaches to understand these interactions. The European Space Agency (ESA)^5^ supports ML model training for real-time diagnostics. Similarly, Polaris Open-Source ML Tool^9^ enhances anomaly detection, including radiation surges during geomagnetic storms. Recent approaches using convolutional autoencoders and LSTM networks^10^ further highlight the potential of unsupervised learning for real-time satellite health monitoring.

EgyptSat-1, the first Egyptian remote sensing satellite, was launched on April 17, 2007, with a 120 kg mass and a 3–5 years lifespan^7^. It marked a significant leap forward in the country’s space exploration efforts. It is equipped with multispectral and infrared imagers. The key Subsystems include Power (PSS), Attitude Determination and Control Subsystem (ADCS), Payload Command and Data Handling Subsystem (PLCDHS), and communication. EgyptSat-1, part of the Egyptian space program dedicated to capacity-building and Earth observation, serves as an ideal candidate for studying these interactions due to its operational timeline and data availability. Designed for a mission duration of at least five years, the satellite was lost just 40 months into operation in 2010. The failure of EgyptSat-1 remains unexplained due to the lack of onboard diagnostic sensors. This limitation has hindered the ability to identify potential causes, including whether external factors such as space weather may have contributed. In this study, we investigate the possible relationship between space weather events and degradation in the satellite’s power subsystem performance by analysing telemetry data alongside external space weather observations.

This study addresses the research question: *Can machine learning–driven frameworks predict and explain the nonlinear impacts of space weather on satellite power systems*,* and thereby detect early signs of subsystem anomalies?* To test this, we formulated two hypotheses. H1: Two stage -based feature extraction captures latent nonlinear dependencies between telemetry and space weather variables better than raw inputs, evaluated via improvements in model accuracy (MAE, RMSE, R²). H2: Deep learning (CNN, LSTM) and ensemble models (Random Forest, AdaBoost, Gradient Boosting, Voting Regressor) outperform baseline statistical correlations in predicting satellite TBS1_Current and NN1_Voltage, evaluated using cross-validated error metrics.

Although prior studies have recognized the role of space weather in satellite anomalies, most rely on descriptive analyses or simple statistical correlations that fail to capture the complex, nonlinear interactions between high-dimensional telemetry data and dynamic environmental conditions. This gap highlights the need for predictive, data-driven frameworks capable of accurately modelling satellite–space weather interactions and detecting early signs of subsystem degradation. To bridge this gap, we introduced a four stages data driven workflow including (1) data preprocessing, (2) to addresses data complexity, a two-stage non-linear feature selection process, initially unsupervised RBM identifies a compact and structurally stable feature subspace. This is followed by a supervised MI validation to guarantee that the final feature set maintains maximum predictive power relative to the specific satellite target variables. (3) leverages six algorithms: CNN, LSTM, Random Forest, Adaptive Boost, Gradient Boosting, and Voting Regressor to model current and NN1_Voltage of Egypt-sat 1. (4) anomaly detection and validation by correlating prediction residuals with space weather disturbances, using STL decomposition and Z-score for GCR and P10 anomaly detection, and coincidence rate analysis to assess temporal alignment. By moving beyond correlation-based methods, this study introduces the first machine learning–driven quantitative analysis of space weather impacts on satellite power systems, advancing operational strategies and supporting the design of more resilient satellite architectures. The key contributions can be summarized:

- Introducing a unified four-stage workflow that systematically integrates data preprocessing, feature learning, predictive modelling, and anomaly validation to study satellite–space weather interactions.
- A two-stage non-linear feature selection process to address data complexity by first using an unsupervised Restricted Boltzmann Machine (RBM) for a compact, stable feature set, followed by supervised Mutual Information (MI) validation to ensure maximum predictive.
- Providing evidence for potential space weather effects on power anomalies via statistically significant coincidence rates, highlighting the need for further cross-system investigations.

## Literature review

### Effect of space weather on satellite performance

Space weather significantly impacts satellite systems, particularly in communication, navigation, and power subsystems^8^. These disturbances—characterized by solar activity, high-energy particle flux, and ionospheric fluctuations—can disrupt satellite functions across orbits. For example, high-energy electrons have been linked to power amplifier failures in geostationary satellites^8^, while phantom commands and spurious signals appear during geomagnetic storms^12^, Navigation systems also experience signal degradation due to ionospheric irregularities and TEC variations^13^.

Power subsystems, particularly solar panels, are highly vulnerable to radiation-induced degradation from solar flares, CMEs, and Galactic Cosmic Rays (GCR). These effects reduce solar cell efficiency^14^, with recent studies proposing material-level innovations and shielding to mitigate damage^15,16^. Notably, geomagnetic storms induce currents that degrade power systems^17^, and long-term GCR exposure accelerates solar panel aging^18^. These findings underscore the need to monitor how external space weather variables influence internal satellite states—insights that can now be enhanced through machine learning (ML). While previous efforts on EgyptSat-1 suggested that extreme space weather events may have played a role in power anomalies^7^, a comprehensive model integrating space weather data and telemetry remained unexplored.

### Machine learning in space weather

Machine learning (ML) has become an essential tool in space weather forecasting, offering advanced capabilities for analyzing complex datasets and enhancing predictive accuracy. Traditional empirical and physics-based models often struggle to capture the nonlinear interactions inherent in solar-terrestrial dynamics. In contrast, ML approaches, including deep learning and ensemble methods, have been increasingly applied to improve space weather forecasting and anomaly detection. For instance, three ML models: Support Vector Machine (SVM), Random Forest (RF), and Light Gradient Boosting Machine (LightGBM)^19^ were utilized to classify solar flares. All models successfully predicted M- and X-class solar flares up to 72 h in advance, achieving a True Skill Statistic (TSS) of 0.58 for 24-hour forecasts. The results obtained highlight the significance of data balancing in enhancing accuracy for space weather events. In^20^, 180-year composite of the geomagnetic *aa* index were used to model geomagnetic activity across solar cycles. By fitting each cycle with a parameterized Gaussian curve, they estimated Solar Cycle 25’s peak aa index at 21 ± 3 nT, suggesting weak activity comparable to Cycles 11 and 13. This highlights the utility of historical data for forecasting geomagnetic trends. The DENSER project demonstrated that neural networks could forecast proton flux and geomagnetic indices^21^. ML has also been used to predict the Bz magnetic field in the early phase of solar storms^22^, and the MAGFiLO dataset was created to integrate solar physics with AI for real-time solar filament detection^23^. These developments illustrate ML’s growing role in space weather forecasting and highlight the potential for integrating environmental drivers with satellite telemetry to predict power anomalies, an area still underdeveloped in existing research.

### Machine learning in satellite telemetry

Various ML approaches have been explored for satellite telemetry prediction. LSTM architecture^24^ was utilized to predict telemetry health parameters during non-visible periods. By capturing long-term dependencies, LSTM models proved effective for anomaly detection and trend forecasting, making them ideal for spacecraft telemetry monitoring. XGBoost model^25^ was applied the to predict the voltage of Amazonia-1 satellite’s Battery Module 1, achieving an R² of 99.99%, with minimal errors. An integration of Wavelet Transforms with Recurrent Neural Networks (RNNs) was introduced for satellite telemetry prediction where the input signals was decomposed using wavelet transforms and used RNNs to predict future wavelet coefficients, improving prediction accuracy and forecast horizons in satellite telemetry^26^.

In addition, MFCA-LSTM model was developed to integrate time- and frequency-domain features with auxiliary information, such as telecommands and mission planning data. This model improves anomaly detection in satellite telemetry by utilizing a two-level framework for comprehensive data analysis, thereby enhancing detection accuracy and reducing false alarms^24^. Hodrick-Prescott (HP) filtering was^27^ utilized to predict satellite telemetry based on time series decomposition using. By separating the telemetry data into trend and fluctuation components, and utilizing polynomial fitting along with seasonal ARIMA models, the method achieved a prediction error of less than 0.67% for satellite solar array current over a one-month period, highlighting the potential of time-series decomposition in precision health monitoring.

Extreme Learning Machine (ELM) optimized with the Grey Wolf Optimization (GWO) algorithm was introduced for anomaly detection^28^. The GWO-ELM model improved anomaly detection efficiency in satellite telemetry data by optimizing the input weights and bias parameters of the ELM, surpassing the performance of both basic ELM and Support Vector Machine (SVM) models. Furthermore, Several ML techniques were compared for predicting satellite telemetry parameters from the EgyptSat-1 satellite. The results confirmed that Long Short-Term Memory (LSTM) and Gated Recurrent Unit (GRU) models performed best in forecasting solar panels temperature, power bus voltage, and total solar panels current, emphasizing the value of deep learning for satellite health monitoring^7^. These studies collectively demonstrate that ML can accurately capture complex temporal dependencies in telemetry data. However, the integration of space weather indicators into telemetry-based models for predicting power subsystem anomalies remains limited, underscoring the need for unified frameworks that combine environmental drivers with satellite health data.

## Materials and methods

### Datasets

#### The space weather dataset

The space weather parameters used to in assessment include solar indices (Sunspot Number (SSN), solar radio flux at a wavelength of 10.7 centimetres (2800 MHz) (F10.7)), geomagnetic indices (planetary geomagnetic index (Kp), the daily index of geomagnetic activity derived from the Kp index (Ap), Disturbance Storm Time (Dst index)), Solar wind properties (density, speed, Latitude (Lat.), Longitude (Long.)) and Galactic Cosmic Rays (GCR) with different channels (PF60, FP30, FP10), Additional parameters comprise auroral indices (Aurora index including (AL), Auroral Upper index (AU), Auroral Electrojet index (AE)), and magnetic field parameters including the components of the Interplanetary Magnetic Field (IMF) measured in the Geocentric Solar Magnetospheric (GSM) coordinate system, Bx, By, Bz. The magnitude of the magnetic field vector often calculated as $\:B=\sqrt{{B}_{x}^{2}+{B}_{y}^{2}+{B}_{z}^{2}}$. The data covering the operational period of EgyptSat-1, (September 9, 2007, to July 17, 2010), corresponding to solar minimum conditions. GCR data were obtained from six globally distributed neutron monitors (Table 1) that record secondary particle counts generated by cosmic ray interactions with the atmosphere, accessible via “http://cr0.izmiran.ru/common/links.htm”.

**Table 1:** The specifications of neutron monitors.

Monitor name	Altitude	Latitude	Longitude	Cutoff rigidity (*R*)	Operation time
MCMURDO	48 m	77.9 S	166.6 E	0.3 GV	11/5/1960 to 7/1/2017
FORTSMITH	180 m	60.02 N	11.93 W	0.3 GV	4/10/2000 to now
NAIN	46 m	56.55 N	61.68 W	0.3 GV	10/11/2000 to now
INUVIK	21 m	6.36 N	133.72 W	0.3 GV	28/09/2001 to now
SWARTHMORE	50 m	39.68 N	75.75 W	2.4 GV	1–7-1964 to now
THULE	26 m	76.5 N	68.7 W	0.3 GV	13/8/1957 to now

Geomagnetic activity was evaluated using the Kp index, from GFZ Potsdam (https://www.gfz-potsdam.de/en/kp-index). Solar wind parameters and additional geomagnetic indices were sourced from NASA’s OMNI Web Data Explorer (https://cdaweb.sci.gsfc.nasa. gov), which provides extensive near-Earth solar wind and magnetic field data, including plasma properties (velocity, density, temperature, and pressure), IMF components, and geomagnetic indices (Kp index). The dataset spans 2007–2010, during solar cycle 23, a solar minimum period when GCR effects are most pronounced. Thus, the dataset comprises 1,043 entries with 27 features, providing a robust framework for analysing space weather impacts on satellite telemetry. Space weather data were resampled to daily resolution, with hourly GCR data averaged to daily values for consistency. The dataset is complete, with no gaps, ensuring reliable long-term analysis of solar and geomagnetic variations.

#### EgyptSat-1 telemetry data

Power Bus Voltage (NN1_Voltage), Total Solar panel Current (TBS1_Current), Solar panel 1 Temperature (T1BS) and Solar panel 3 Temperature (T3BS) were obtained from the Egyptian Space Agency (EgSA) for time span between September 2007 to November 2010. To ensure the conversion of raw telemetry data into accurate physical measurements, precise sensor calibration was employed using Eq. (1):

1 $$\:Analog\:Sensor\:Value\:=\:\left(code\:X\:0.03\right)-\:0.98$$

where ‘code’ represents the telemetry data in decimal form.

The satellite telemetry data, sampled at one-second intervals with ~ 10% missing values, were resampled to daily resolution for consistency with space weather data while preserving key variability. This preprocessing reduced high-frequency noise and highlighted long-term trends. Mean NN1_Voltage and TBS1_Current were 31.02 V and 6.3 A, respectively, indicating baseline power subsystem performance. to ensure that the analysis focuses on periods when the satellite’s solar panels were illuminated and thus more likely to be directly impacted by space weather events, only the maximum values of Solar Panel 1 Temperature (T1BS) and Solar Panel 3 Temperature (T3BS) were considered. These maximum readings, ranging from − 10 °C to + 45 °C, correspond to times when the panels were facing the Sun, maximizing exposure to potential radiation-induced effects. A comprehensive description of the EgyptSat-1 telemetry data processing methodology is provided in. Table 2 summarises the complete dataset which consists of 27 features, including 23 space weather parameters and the remaining 4 are telemetry variables.

**Table 2:** Summary of the complete dataset comprising space weather and EgyptSat-1 telemetry parameters (2007–2010).

Category	Parameter	Unit	Source/Instrument	Temporal Resolution
**Satellite Telemetry**	NN1_Voltage	Volt (V)	EgyptSat-1 onboard power subsystem (bus voltage sensor)	1-s raw → daily mean
	TBS1_Current	Ampere (A)	EgyptSat-1 onboard power subsystem (solar panel current sensor)	1-s raw → daily mean
	T1BS	°C	EgyptSat-1 onboard thermal sensor (solar panel 1)	1-s raw → daily mean
	T3BS	°C	EgyptSat-1 onboard thermal sensor (solar panel 3)	1-s raw → daily mean
**Geomagnetic Indices**	Kp	Dimensionless (0–9)	GFZ Helmholtz Centre for Geosciences https://kp.gfz.de/en/	3-hourly → daily mean
	F10.7	Solar Flux Unit (sfu)	**Natural Resources Canada/NOAA** https://www.spaceweather.gc.ca/forecast-prevision/solar-solaire/flux/sx-5-en.php	Daily mean
	ap	nT	GFZ Helmholtz Centre for Geosciences https://kp.gfz.de/en/	3-hourly → daily mean
	AE	nT	NASA OMNIWeb (Kyoto WDC data included) https://omniweb.gsfc.nasa.gov/form/dx1.html	1-min → daily mean
	AL	nT	NASA OMNIWeb (Kyoto WDC data included) https://omniweb.gsfc.nasa.gov/form/dx1.html	1-min → daily mean
	AU	nT	NASA OMNIWeb (Kyoto WDC data included) https://omniweb.gsfc.nasa.gov/form/dx1.html	1-min → daily mean
**Solar & Particle Parameters**	SSN (Sunspot Number)	Dimensionless	GFZ Helmholtz Centre for Geosciences https://kp.gfz.de/en/	Daily
	F10.7 (Solar Radio Flux)	sfu (10⁻²² W m⁻² Hz⁻¹)	GFZ Helmholtz Centre for Geosciences https://kp.gfz.de/en/	Daily
	P10, P30, P60 (Proton Fluxes)	pfu (cm⁻² s⁻¹ sr⁻¹)	NASA OMNIWeb (NOAA GOES satellites) https://omniweb.gsfc.nasa.gov/form/dx1.html	5-min → daily mean
**Cosmic Rays**	GCR (Galactic Cosmic Rays)	counts hourly	Six globally distributed neutron monitors (see Table 1); data via *IZMIRAN NMDB* http://cr0.izmiran.ru/common/links.htm	Hourly → daily mean
**Solar Wind & IMF**	Solar Wind Speed	km/s	NASA OMNIWeb (ACE/DSCOVR spacecraft) https://omniweb.gsfc.nasa.gov/form/dx1.html	1-min → daily mean

#### Proposed workflow

**Fig. 1:**
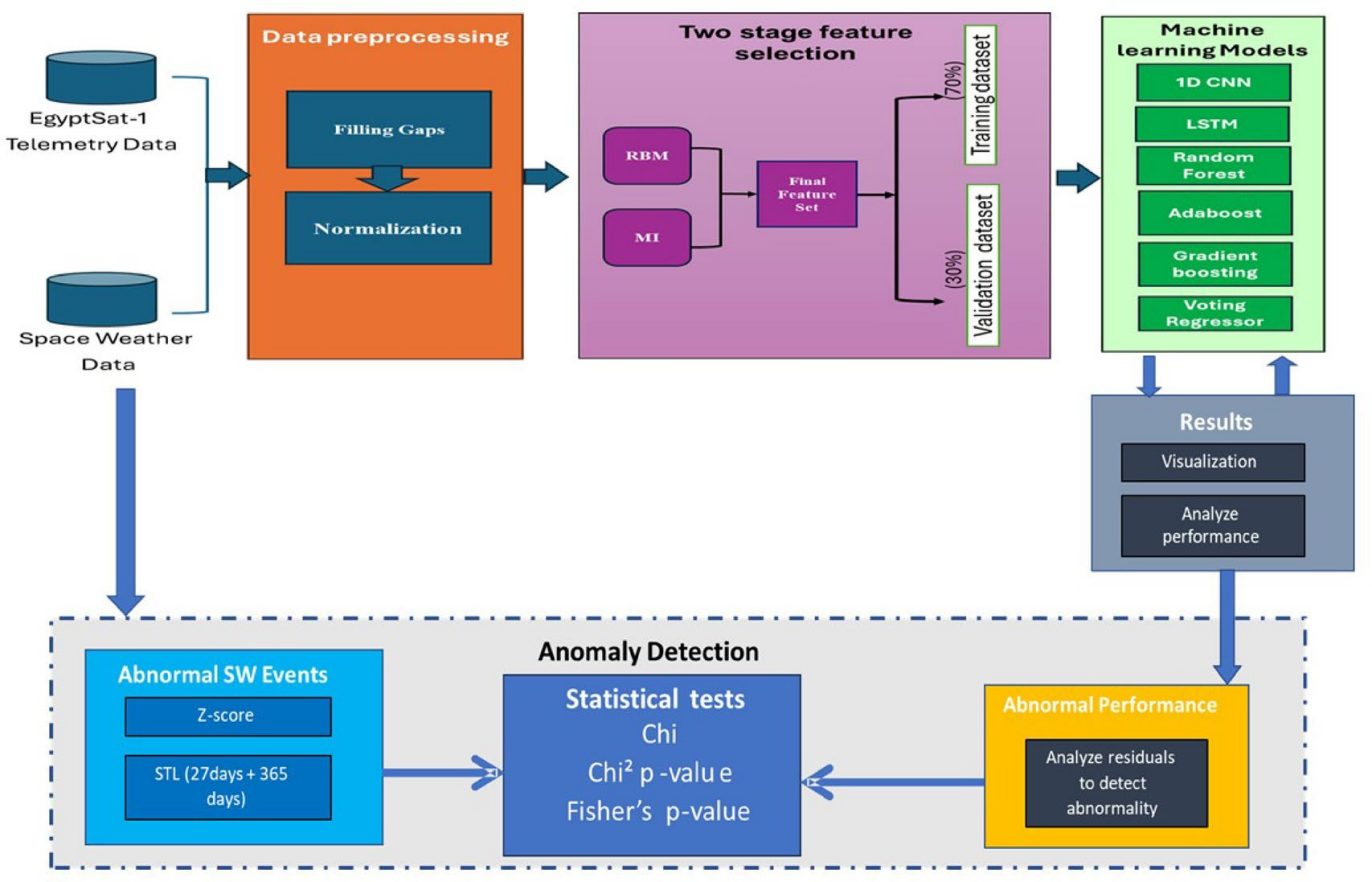
The proposed workflow for investigating the impact of weather parameters on satellite power subsystem, focuses on EgyptSat-1.

The proposed data-driven workflow to analyse the impact of weather conditions on EgyptSat-1 NN1_Voltage and TBS1_Current, as shown in Fig. 1. It comprises of four main stages, data preprocessing, feature selection, machine learning models, and abnormality validation. Initially, data preprocessing addresses missing values to ensure data quality. Two stage Feature selection was performed, first an unsupervised Restricted Boltzmann Machine (RBM) was utilized to identify a compact, structurally stable feature set, followed by supervised Mutual Information (MI) validation for maximum predictive relevance to the satellite targets (T1BS, T3BS). Six Machine Learning (ML) models namely: Convolutional Neural Networks (CNN), Long Short-Term Memory (LSTM) networks, Random Forest Regressor, Adaptive Boosting, Gradient Boosting and Voting Regressor, were incorporated to handle dynamic and complex nature of telemetry and weather datasets, with performance evaluated using metrics such as Mean Absolute Error (MAE), Mean Squared Error (MSE), Root Mean Squared Error (RMSE) and Mean Absolute Percentage Error (MAPE). Finally, the anomaly detection and validation stage identify deviations between predicted and actual power telemetry values (residuals). Anomalies in GCR and P10 were detected using STL decomposition followed by Z-score analysis on the residuals. This approach isolates transient space weather effects. Detected anomalies were validated by assessing coincidence with residual anomalies in NN1_Voltage and TBS1_Current, highlighting potential space weather impacts. The proposed workflow includes the following stages:

1. **Data Pre-Processing**.

The space weather and telemetry data were pre-processed through gap filling and normalization. Missing values were imputed using forward and backward filling to preserve temporal continuity. Min-max normalization was then applied to standardize feature scales, enabling consistent comparison and reducing unit-based bias in subsequent analyses.

(2) **Two-Stage Non-linear Feature Selection**.

To manage the high dimensionality, multicollinearity, and non-linear nature of the 23 space weather input features, we implemented a two-stage non-linear feature selection method to transforms the input space into a compact, robust feature set.

***Stage 1: Restricted Boltzmann Machine (RBM)***: a specialized form of the Boltzmann Machine, is employed as the initial, unsupervised stage of our feature selection process. Its purpose is to learn a highly compact, non-linear latent representation from the 23 space weather input data. The RBM consists of two layers: a visible layer that represents the input features (the space weather indices) and a hidden layer designed to capture underlying, non-linear dependencies. Unlike general Boltzmann Machines, RBMs^30^ have no intra-layer connections, with each unit in one layer fully connected to all units in the other layer. The RBM is trained exclusively on the space weather inputs, optimizing its weights and biases, as defined in Eq. (2) to learn a probability distribution that efficiently reconstructs the input data.

2 $$\:E\left(x,h\right)=-{h}^{T}Wx-{c}^{T}x-{b}^{T}h=-\sum\:_{j}\sum\:_{k}{W}_{j,k}{h}_{j}{x}_{k}-\sum\:_{k}{c}_{k}{x}_{k}-\sum\:_{j}{b}_{j}{h}_{j}$$

where, X_k_, h_j_ represent visible and hidden layer units, respectively. W_j, k_ represents weight between layers and c_k_, b_j_ are bias terms.

Crucially, the RBM weights are interpreted as indicators of structural importance, not predictive influence. The magnitude of a feature’s total outgoing weight reflects its contribution to the entire learned latent space, a measure of its stability and robustness across the input domain. This process effectively isolates the features that contain the most significant non-linear variance and are the least redundant, which are then passed to the subsequent supervised Mutual Information method for validation against the output target.

Stage 2: Supervised Validation via Mutual Information (MI): To link the features pre-selected by the RBM to the supervised target variable (NN1_Voltage, TBS1_Current), we introduced a second, supervised validation step using Mutual Information (MI).

- The MI was computed between each RBM-selected feature and target variable (NN1_Voltage, TBS1_Current).
- This procedure is designed to ensure that the features previously selected for their structural stability (Stage 1) also possess the maximum informational value and predictive efficacy required for accurately forecasting power anomalies within the satellite system.
- Only features confirmed by both the RBM (structural significance) and the MI (predictive relevance) were passed forward to the next step.

(3) **Machine Learning Models**.

Six machine learning models namely: CNN, LSTM, Random Forest, AdaBoost, Gradient Boosting, and Voting Regressor, were used to predict satellite NN1-Voltage and TBS1_Current, with hyperparameters optimized via cross-validation.

***A Convolutional Neural Network (CNN)*** A CNN is a deep learning architecture that applies convolution operations to sequential data, enabling the extraction of local features and hierarchical representations. In its one-dimensional (1D) form, convolutional filters operate along the temporal axis, making CNNs particularly effective for capturing localized temporal dependencies in satellite telemetry and space weather time series.

***Long Short-Term Memory Networks (LSTM)*** are a type of recurrent neural network (RNN) designed to overcome vanishing and exploding gradient problems in long sequences. They employ memory cells and gating mechanisms (input, forget, and output gates) to capture both short- and long-term dependencies. This makes LSTMs well suited for modeling the nonlinear temporal dynamics inherent in satellite power performance data^31^.

***Random Forest Regressor (RF)***^32^ is an ensemble learning method that constructs multiple decision trees and aggregates their outputs to improve predictive accuracy and robustness. By combining randomized feature selection and bootstrap aggregation (bagging), RF reduces overfitting and effectively captures nonlinear relationships between predictors and target variables in high-dimensional datasets.

***Adaptive Boosting (AdaBoost)***^33^ is an ensemble method that iteratively trains a sequence of weak learners, typically shallow decision trees, by reweighting misclassified samples to focus on difficult cases. Through this adaptive reweighting mechanism, AdaBoost progressively enhances predictive performance while maintaining relatively low variance, making it effective for complex regression tasks such as anomaly detection in satellite telemetry.

***Gradient Boosting (GBM)***^34^ is an ensemble method that sequentially adds weak learners to minimize prediction errors by fitting the negative gradient of the loss function. At each stage, new models are trained to approximate the negative gradient of the loss function, thereby reducing prediction errors. This approach allows GBM to model complex nonlinear dependencies and achieve high predictive accuracy in regression tasks involving satellite and space weather data.

***Voting Regressor***^35^ is an ensemble learning model used to combine multiple regression models and improve prediction accuracy. It aggregates the predictions from multiple base models using a hard voting strategy with uniform weights confidence scores, to produce a final robust prediction.

(4) **Abnormality Detection**.

Anomalies in the satellite power subsystem were identified by quantifying significant deviations between model predictions and actual telemetry observations. Predicted values were obtained using the best-performing machine-learning model—Random Forest (RF)—trained on the selected space-weather features. Residuals were computed as the difference between predicted and observed values, and an observation was flagged as anomalous when the residual exceeded ± 1.5 σ from the residual distribution mean. This threshold provided an optimal balance between sensitivity to rare events and suppression of false positives, with particular emphasis on the critical telemetry variables NN1_Voltage and TBS1_Current.

For space weather parameters, anomaly detection was performed using both Z-score analysis and Seasonal-Trend Decomposition using Loess (STL)^36^. The Z-score^37^ is a statistical metric that measures how many standard deviations a value deviates from the dataset mean and is expressed as

3 $$\:Z=\frac{X-\mu\:}{\sigma\:}$$

where $\:X$ is the data point. µ, and σ are the mean and standard deviation of the dataset. In this study, anomalies were defined as events where the Z-score exceeded ± 1.5σ. a threshold physically motivated by the deep solar minimum (2007–2010), when GCR fluxes were anomalously high and proton fluxes elevated, requiring a more permissive cutoff to capture meaningful variability.

*Decomposition using Loess (STL*)^36^ decomposition was applied to break down the time-series data into three components: trend, seasonal, and residual:

4 $$\:y\left(t\right)=T\left(t\right)+S\left(t\right)+R\left(t\right)$$

where T(t), S(t), and R(t) are the trend, seasonal and residual components.

This approach effectively separated the 27-day solar-rotation and 365-day annual periodicities from transient disturbances. The residual component was then analysed using the Z-score criterion, enabling detection of both short-term fluctuations (e.g., solar proton enhancements, Forbush decreases) and long-term deviations linked to solar-cycle variations. The combined STL + Z-score framework thus captured anomalies across multiple temporal scales with improved robustness.

Feature-weighting analysis using the two-stage feature selection was further employed to evaluate the relative contribution of space-weather drivers. two-stage feature selection assigned the highest weights to the GCR counts and P10 proton flux (≥ 10 MeV), identifying these as the dominant environmental parameters influencing satellite power-system anomalies.

Finally, anomalies in space weather parameters were statistically compared with those identified in satellite telemetry. A ± 3-day coincidence window was applied to capture potential time lags between space weather disturbances and satellite subsystem responses. Statistical validation was conducted using Chi-square tests^38^ of independence and Fisher’s exact test^39^.

## Experimental results and discussion

### Experimental setting

To ensure a fair comparison, space weather and telemetry time-series datasets spans 09 September 2007–17 July 2010. A chronological split was applied: training (70%) from 09 September 2007–06 September 2009 (729 days) and testing (30%) from 07 September 2009–17 July 2010 (313 days). Normalization was fitted on the training set only, and time-series cross-validation used forward-chaining folds to avoid temporal leakage. Temporal dependencies were evaluated using 1, 2, and 3, 4,5 step lags. To ensure all models had equivalent access to temporal context, enabling a fair comparison, sequential models (CNN, LSTM) were trained with multi-day input sequences using a 3-day sliding window to directly capture inherent temporal dependencies. On other hand, Tabular Regressors (RF, GBM, AdaBoost) were trained with lag-expanded static features corresponding to the same 3-day look-back horizon.

Hyperparameter tuning was conducted via grid search using a time-series walk-forward validation framework, evaluated through 5-fold time-series cross-validation. This approach ensured that the temporal order of observations was maintained and that no future information leaked into model training. The detailed search spaces and the corresponding optimal configurations for each model are summarized in Table 3. Following the selection of the optimal hyperparameters, the final model was retrained on the entire training dataset to maximize predictive capacity before being evaluated once on the independent test set.

**Table 3:** Hyperparameter search space and selected best configurations for machine learning models (seed = 42).

Model	Search grid (key params)	Selected Architecture/Parameters	Training Setup
**1D-CNN**	filters ∈ {16, 32, 64}; kernel ∈ {3, 5}; conv blocks ∈ {1, 2}; dropout ∈ {0, 0.2};batch ∈ {32, 64}; lr ∈ {1e-4, 1e-3, 1e-2}	1 convolutional block (32 filters, kernel = 3), dropout = 0.2, max pooling, fully connected layer (32 neurons)	Adam (lr = 1e⁻³), batch = 32, epochs = 81, RMSE loss
**LSTM**	hidden ∈ {32, 64, 128}; layers ∈ {1, 2}; dropout ∈ {0, 0.2,0.5}; batch ∈ {32, 64}; lr ∈ {1e-4, 1e-3, 1e-2}	1 LSTM layer (64 units), dropout = 0.2, FC layers: 32 → 1	Adam (lr = 1e⁻³), batch = 32, epochs = 73 (early stopping), RMSE loss
**Random Forest (RF)**	n_estimators ∈ {200, 400, 800}; max_depth ∈ {None, 8, 16}; min_samples_leaf ∈ {1, 2, 4}	n_estimators = 400, max_depth = None, min_samples_leaf = 2	Tree-based ensemble averaging
**Gradient Boosting (GBM)**	n_estimators ∈ {100, 200, 400, 800}; learning_rate ∈ {0.01, 0.05, 0.1}; max_depth ∈ {2, 3, 4}; subsample ∈ {1.0, 0.8}	n_estimators = 100, max_depth = 3, learning_rate = 0.05	RMSE loss function
**AdaBoost**	n_estimators ∈ {200, 400, 800}; learning_rate ∈ {0.01, 0.05, 0.1}; base_estimator = DecisionTree(max_depth ∈ {2, 3, 4})	n_estimators = 400, learning_rate = 0.05, base_estimator = DecisionTree(max_depth = 3)	RMSE loss function
**Voting Regressor**	estimators ∈ subset of {LSTM, CNN, RF, GB, Ada}; weights tuned on val ∈ {[1,1,1], [2,1,1], [1,2,1], {2,2,1}}	Combined models: RF, GBM, LSTM; weights {2,2,1}	Ensemble aggregation

All six models were evaluated using Mean Absolute Error (MAE), Mean Squared Error (MSE), Root Mean Squared Error (RMSE), and Mean Absolute Percentage Error (MAPE) as defined in Table 4, where $\:{y}_{i}$ represents the actual values, $\:{\widehat{y}}_{i}$ represents the predicted values, and n denotes the total number of observations.

**Table 4:** The four adopted evaluation metrics.

Mean Absolute Error (MAE)	$\:MAE=\:\frac{1}{n}{\sum\:}_{i=1}^{n}\left|{y}_{i}-{\widehat{y}}_{i}\right|$
Mean Squared Error (MSE)	$\:MSE=\frac{1}{n}{\sum\:}_{i=1}^{n}{\left({y}_{i}-{\widehat{y}}_{i}\right)}^{2}$
Root Mean Squared Error (RMSE)	$\:RMSE=\sqrt{MSE}$
Mean Absolute Percentage Error (MAPE)	$\:MAPE=\frac{100\%}{n}{\sum\:}_{i=1}^{n}\left|\frac{{y}_{i}-{\widehat{y}}_{i}}{{y}_{i}}\right|$

## Results

**Fig. 2:**
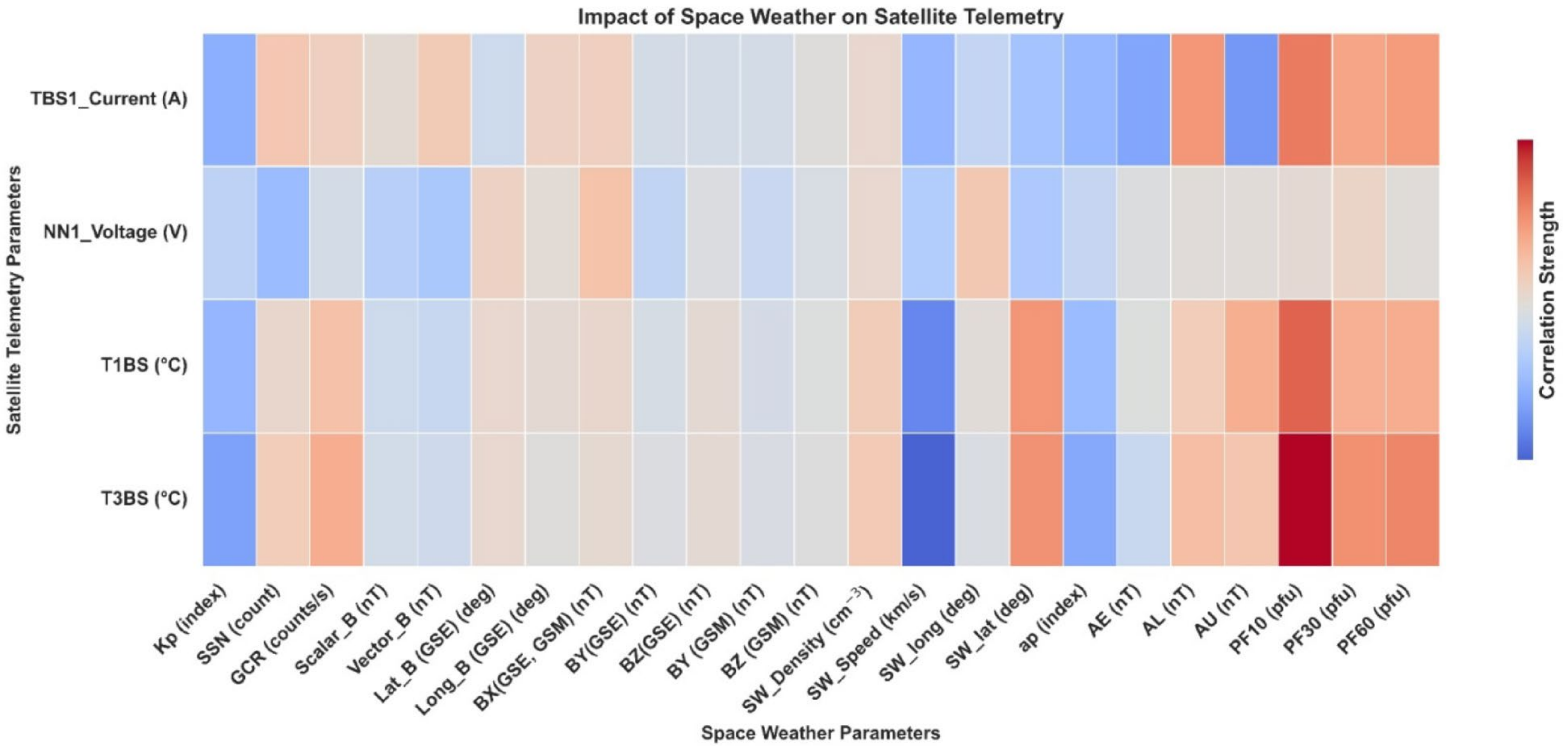
A Correlation heatmap between daily satellite telemetry parameters (vertical) and space weather indices (horizontal). Warmer colors indicate stronger positive correlations, while cooler colors indicate negative correlations.

We conducted various sets of experiments to verify the capability of the proposed workflow. Firstly, Fig. 2 illustrates the correlation heatmap between daily-averaged satellite telemetry parameters and various space weather indices. The telemetry parameters analysed NN1_Voltage, TBS1_Current, and T1BS and T3BS. The space weather drivers considered geomagnetic indices (Kp, ap, AE, AL, AU), (GCR), solar wind parameters, and solar proton fluxes (P10, P30, P60), and the F10.7 solar radio flux. Notably, the heatmap reveals relatively stronger correlations between space weather indices and solar panel-related telemetry (TBS1_Current, T1BS and T3BS temperature) compared to NN1_Voltage, suggesting increased sensitivity of these components to variations in the external space environment. Moderate correlations are observed between telemetry parameters and solar proton fluxes, F10.7, and solar wind characteristics, although these relationships warrant further statistical validation to assess their significance. As expected, a strong correlation is evident between the two solar panel temperature channels (T1BS and T3BS), reflecting their similar physical and operational characteristics.

This aligns with prior studies suggested that space weather disturbances can exert both immediate and cumulative effects on spacecraft systems^12^. Specifically, geomagnetic storms can induce Geomagnetically Induced Currents (GICs), affecting power distribution, while SEP events contribute to radiation damage in solar cells, leading to long-term power degradation^40^.

In the second stage of the proposed framework, the most influential space-weather parameters affecting satellite power telemetry were identified. To achieve this, four feature-importance techniques were applied: the proposed two-stage feature selection, SHapley Additive exPlanations (SHAP), Permutation Importance, and Mutual Information. These methods were selected because they provide complementary analytical perspectives. SHAP quantifies the contribution of each feature to model predictions, Permutation Importance evaluates the change in performance when feature values are randomly shuffled, Mutual Information measures statistical dependency between input variables and target outputs, while the proposed two-stage feature selection not only uses unsupervised Restricted Boltzmann Machine (RBM) for a compact, stable feature set, but also adopted supervised Mutual Information (MI) validation to maximize predictive relevance for the satellite targets.

**Fig. 3:**
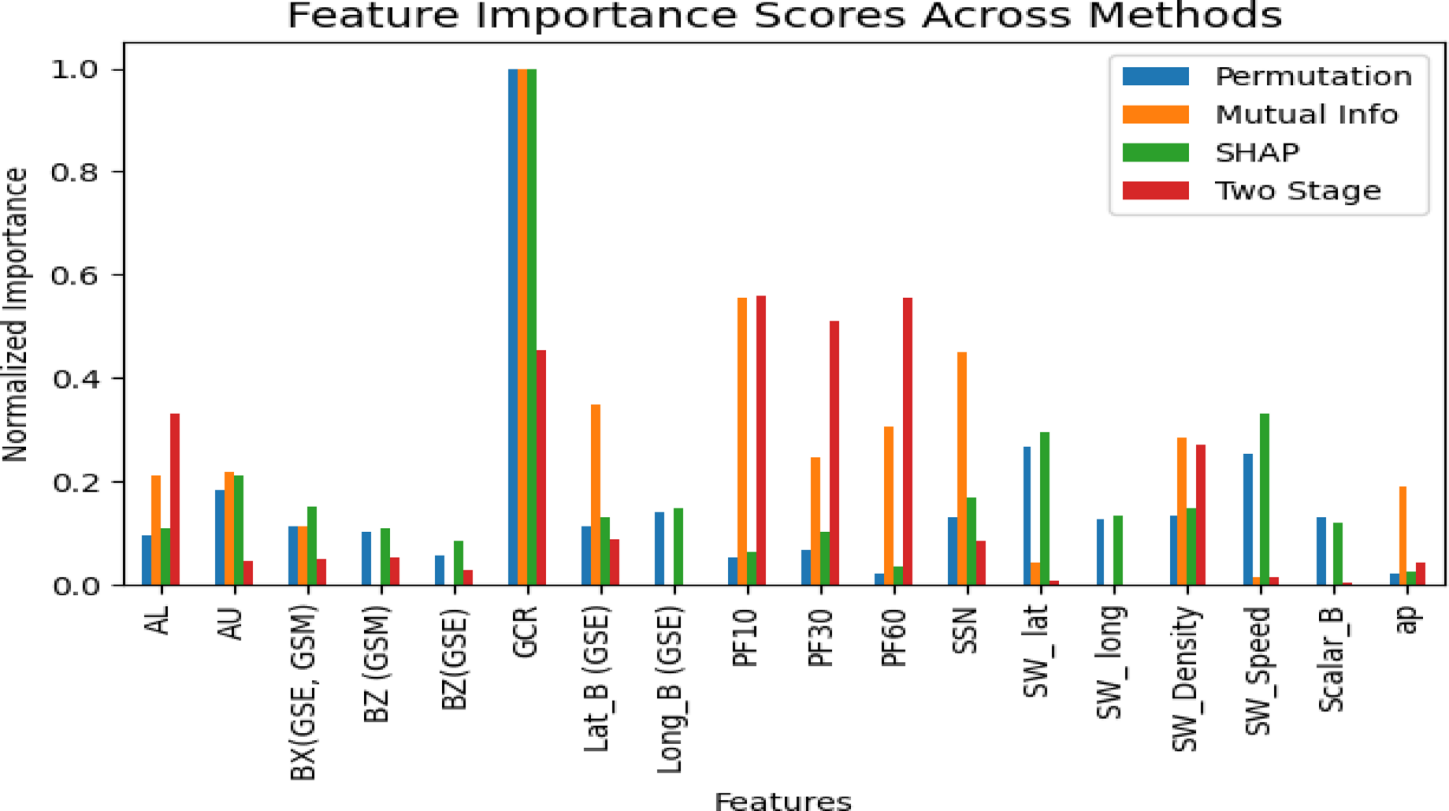
Comparison of top 10 features by two-stage selection, permutation, mutual information, and SHAP methods.

As shown in Fig. 3 and summarized in Table 5, the proposed selection method achieved the lowest error metrics and produced a more compact, stable feature subset than other methods. By learning nonlinear representations, the two-stage approach effectively reduced redundancy and noise, improved generalization, and maintained the physical interpretability of variables under varying space-weather conditions. Overall, it offered the best balance between predictive accuracy and scientific relevance, providing a solid foundation for subsequent anomaly detection in the satellite power subsystem.

The two-stage feature selection effectively extracted latent features, with learned weights highlighting the relative influence of key drivers of power anomalies, as shown in Fig. 3. Dominant features included galactic cosmic rays (GCR), proton flux levels (> 60, > 30, >10 MeV), the AL index, and solar wind density. The strong contribution of proton flux supports prior findings on GCR and SEP degrading spacecraft electronics and solar panels^41,42^. A notable strong contribution of GCR intensity, aligns with well-established space weather phenomena. During solar minimum periods, the reduced solar wind pressure and weakened heliospheric magnetic field allow greater penetration of GCR particles into Earth’s vicinity, increasing their potential impact on satellite electronics^43^. Similarly, the prominence of the AL index and solar wind density underscores the role of auroral activity and solar wind variations in spacecraft charging events^12^.

**Table 5:** Comparison between the performance of top 10 features by Two-Stage feature selection, Permutation, mutual Information, and SHAP methods using RF model.

Features	MSE	RMSE	MAE	MAPE
All 23 feature	0.0165	0.1293	0.0952	0.00322
Top 10 features from proposed two stage feature selection (RBM◊MI)	0.0010	0.0113	0.0053	0.00020
Top 10 feature Permutation^44^	0.0120	0.1087	0.0818	0.00264
Top 10 FeaturesMutual Information^45^	0.0026	0.0118	0.0073	0.00022
Top 10 Features SHAP	0.0031	0.0142	0.0092	0.00029

In the third stage of the proposed framework, six machine-learning (ML) models were developed to predict NN1_Voltage and TBS1_Current of EgyptSat-1 using the ten most influential space-weather features identified in the previous stage. To account for the time-delayed effects of space weather on satellite performance, several lag depths (1–5 days) were tested, following studies reporting that disturbances from galactic cosmic rays (GCRs) and solar energetic protons can influence satellite telemetry with delays of one to three days due to cumulative charging and displacement-damage processes^43^.

**Fig. 4:**
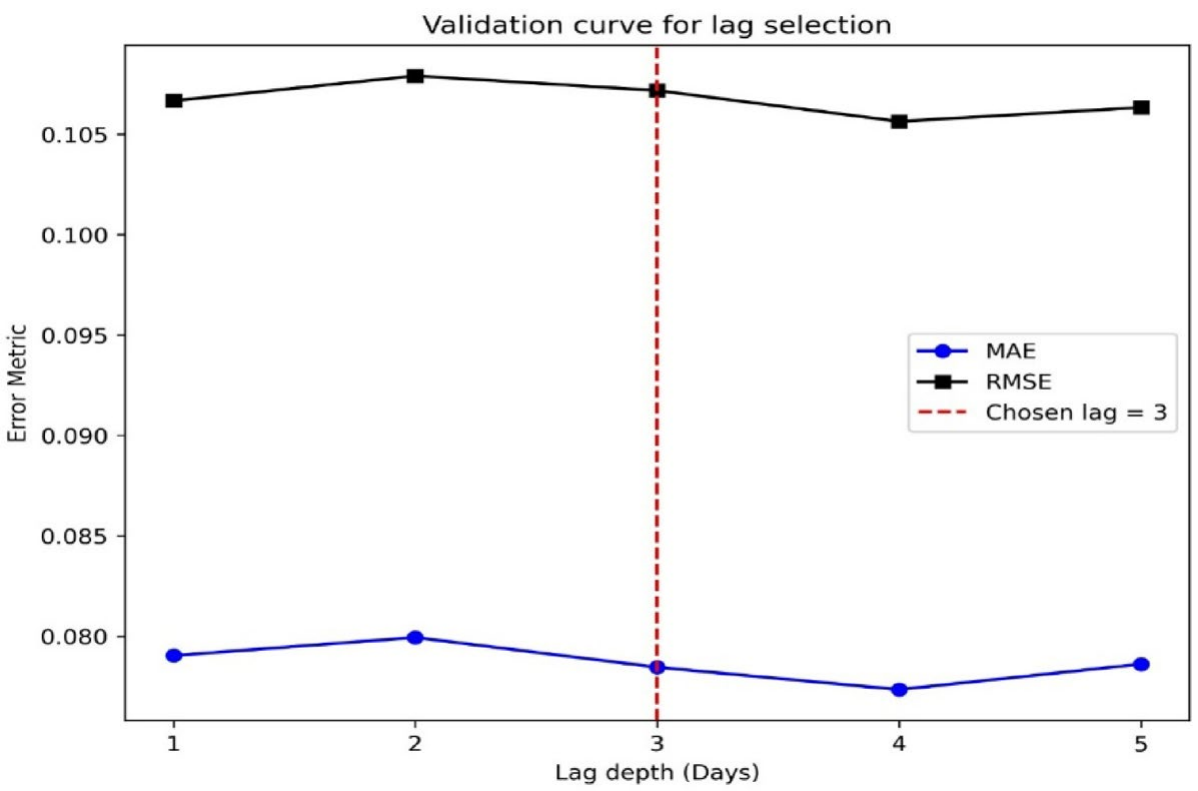
Validation curves for lag-depth selection, showing MAE and RMSE across time-step lags; optimal lag = 3 indicated by dashed line.

The validation curves in Fig. 4 show the variation of MAE and RMSE with different lag depths. Although a lag of four days produced slightly lower error metrics, a lag = 3 days was selected as optimal. This selection aligns with the physical interpretation of solar-wind propagation and magnetospheric coupling timescales described in ESA’s Space Environment Effects Handbook^45^ and empirical studies on LEO spacecraft response to geomagnetic activity^20^. Thus, lag = 3 achieves a realistic balance between predictive accuracy and physical plausibility, reflecting the typical temporal memory between external disturbances and onboard power-system responses.

**Fig. 5:**
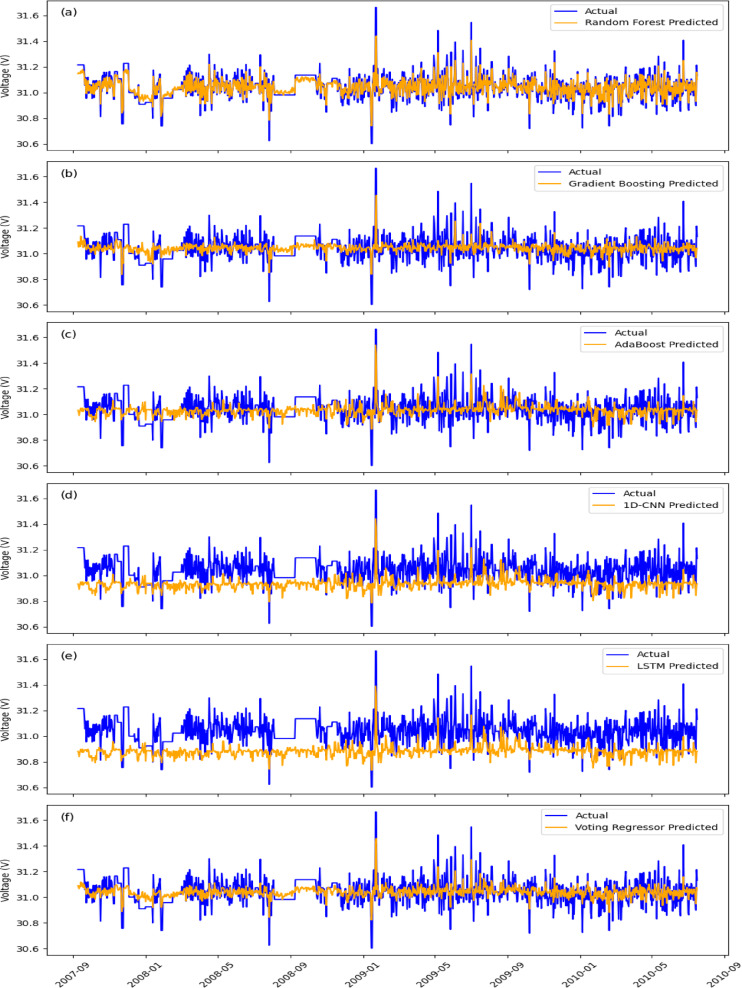
The actual NN1_Voltage (blue) compared against the predicted values (orange) for six machine learning models.

For EgyptSat-1 NN1_Voltage prediction, RF (Fig. 5a) outperformed other models, achieving the lowest MSE (0.001469) and MAPE (0.000909). However, its averaging mechanism limited sensitivity to rapid voltage fluctuations. GBM (Fig. 5b) performed well (MSE: 0.006359, MAPE: 0.001929) but was more sensitive to noise. AdaBoost (Fig. 5c) and 1D-CNN (Fig. 5d) struggled with sharp fluctuations, yielding higher errors (MSE: 0.009049 and 0.063749). LSTM (Fig. 5e) was less effective (MSE: 0.010219, MAPE: 0.002375) due to noisy data. The Voting Regressor (Fig. 5f), combining RF, GBM, and AdaBoost, offered better stability but remained sensitive to extreme deviations.

**Fig. 6:**
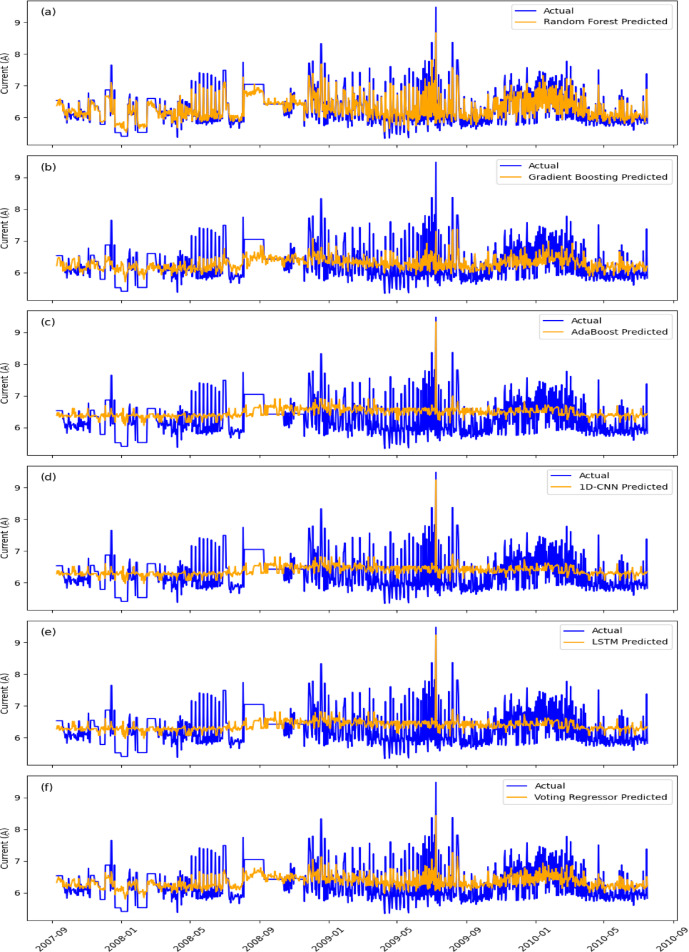
The actual TBS1_Current (blue) compared against the predicted values (orange) for six machine learning models.

For EgyptSat-1 TBS1_Current prediction, RF again outperformed the others (Fig. 6a) with the lowest MSE (0.040499) and MAPE (0.02399). GBM (Fig. 6b) also performed well, but showed more noise sensitivity (MSE: 0.178751, MAPE: 0.050797). AdaBoost (Fig. 6c) and 1D-CNN (Fig. 6d) struggled with fluctuations (MSE: 0.291096 and 0.311908). LSTM (Fig. 6e) was less effective due to the noisy nature of the data (MSE: 0.319108, MAPE: 0.075326). The Voting Regressor (Fig. 6f) improved prediction accuracy by combining multiple models, offering enhanced stability and overall performance.

While these results highlight the utility of tree-based and ensemble approaches, the models still showed limited sensitivity to short-lived anomalies and struggled with noisy TBS1_Current data. Future work could integrate advanced hybrid approaches, such as physics-informed neural networks, causal inference frameworks, or anomaly-aware deep learning models (e.g., variational autoencoders or attention-based architectures), to overcome these limitations and improve generalizability across different satellites and space weather conditions^46–48^.

To sum up, the Random Forest (RF) model exhibited stable performance and lower variability due to its ensemble architecture. In contrast, other models, limited by narrower hyperparameter tuning ranges, may have converged to suboptimal local minima rather than achieving their theoretical global optima. As indicated in Table 3, the hyperparameter optimization process was constrained by computational resources. Consequently, the search space for most models was restricted to a feasible subset of parameters to enable consistent and comparable evaluation across multiple architectures, including CNN and LSTM networks, using extensive space weather dataset.

Table 6 compares model performance on test set is compared using MAE, MSE, RMSE, and MAPE, computed in the original (unscaled) feature space. While the Voting Regressor generally provides balanced and competitive performance by combining the strengths of individual models, it does not consistently outperform all other models across every metric and target variable. Tree-based models (RF, GB) achieved the lowest NN1_Voltage prediction errors, whereas CNN and LSTM performed worse, particularly for TBS1_Current. The ensemble model (Voting Regressor) provided balanced results, reducing NN1_Voltage prediction errors (MSE < 0.01, MAPE < 0.01) and moderately improving TBS1_Current prediction (MSE ≈ 0.15, MAPE ≈ 0.05). These results suggest that tree-based and ensemble models are better suited for NN1_Voltage, while TBS1_Current prediction remains more complex.

**Table 6:** Performance metrics of six machine learning models on NN1_Voltage and TBS1_Current prediction using Hold-Out test set (Original Units).

Model	NN1_Voltage				TBS1_ Current			
	Metric	Mean	Lower	Upper	Mean		Lower	Upper
AdaBoost	MAE	0.074844	0.069339	0.08039	0.463812		0.417505	0.507588
	MAPE	0.002411	0.002235	0.002589	0.074702		0.067108	0.082324
	MSE	0.009049	0.007916	0.01032	0.291096		0.243759	0.339896
	RMSE	0.095074	0.088971	0.101587	0.539046		0.49372	0.583006
1D-CNN	MAE	0.209613	0.190549	0.232345	0.398551		0.345759	0.446801
	MAPE	0.006758	0.00614	0.007493	0.060087		0.052976	0.066117
	MSE	0.063749	0.053554	0.074883	0.311908		0.233235	0.395825
	RMSE	0.252267	0.231418	0.273647	0.557349		0.482944	0.629147
Ensemble	MAE	0.053079	0.048968	0.056971	0.307508		0.274908	0.341264
	MAPE	0.00171	0.001577	0.001834	0.04877		0.043569	0.054106
	MSE	0.004754	0.004057	0.00549	0.140235		0.11536	0.166864
	RMSE	0.0689	0.063697	0.074093	0.374052		0.339646	0.40849
GB	MAE	0.059896	0.055566	0.064453	0.324971		0.287512	0.36334
	MAPE	0.001929	0.00179	0.002076	0.050797		0.045356	0.05658
	MSE	0.006359	0.005368	0.0045	0.178751		0.140968	0.221049
	RMSE	0.079669	0.073269	0.086315	0.422142		0.375457	0.470159
LSTM	MAE	0.073744	0.068253	0.080061	0.469461		0.421585	0.514789
	MAPE	0.002375	0.002199	0.002579	0.075326		0.067283	0.083435
	MSE	0.010219	0.008249	0.01297	0.319108		0.265692	0.383182
	RMSE	0.100896	0.090823	0.113886	0.564198		0.515453	0.619017
RF	MAE	0.02821	0.026154	0.030539	0.153269		0.13571	0.168704
	MAPE	0.000909	0.000842	0.000983	0.02399		0.021456	0.026197
	MSE	0.001469	0.001197	0.001835	0.040499		0.031948	0.048949
	RMSE	0.038263	0.034595	0.042837	0.200942		0.17874	0.221245

**Fig. 7:**
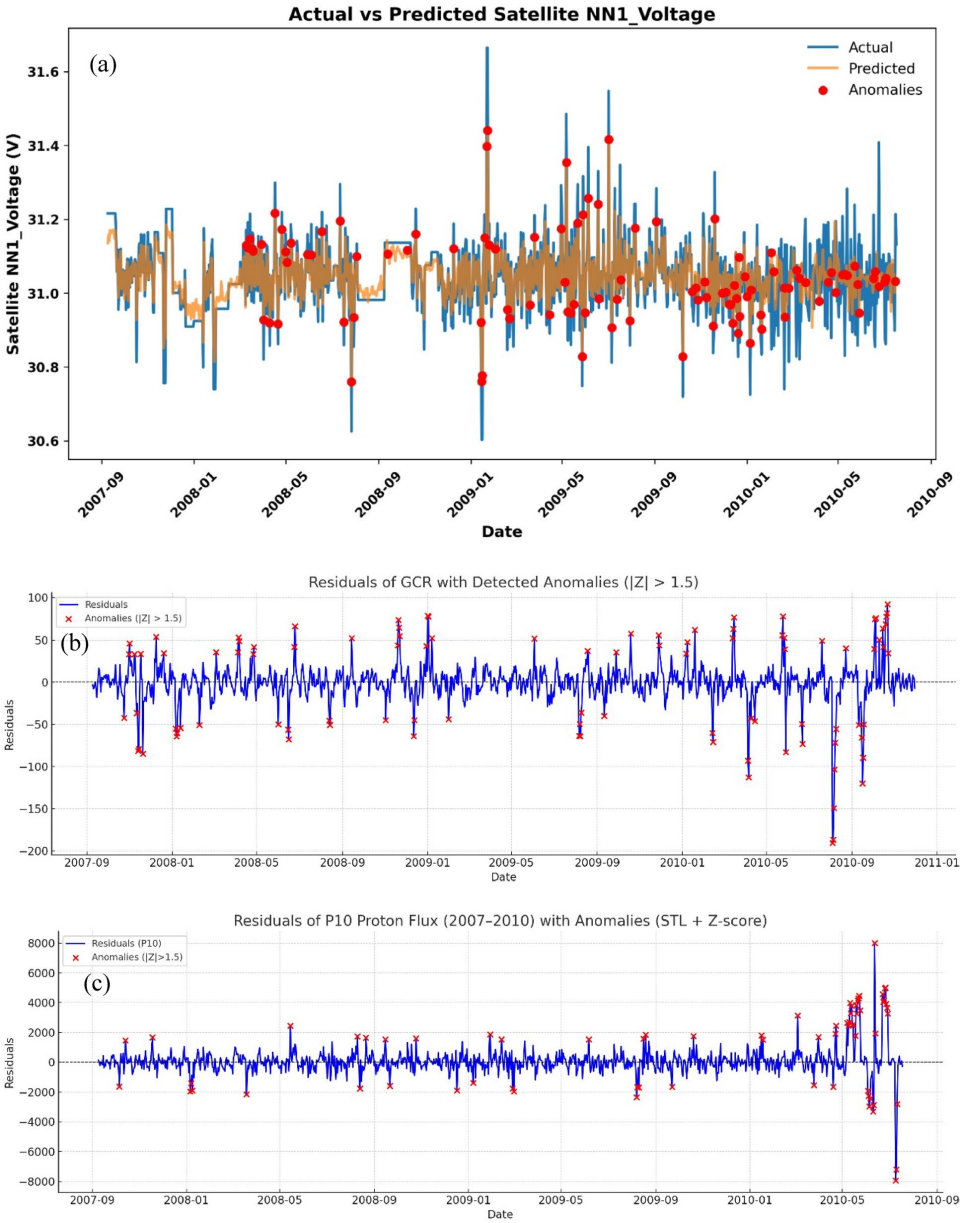
(**a**) The voltage anomaly predicted using RF model anomalies where the residual (actual – predicted) exceeds ± 1.5 standard deviations (σ). (**b**)GCR residuals with anomalies (|Z| > 1.5) marked in red, showing fluctuations over 2007–2010. (**c**) Residuals of P10 proton flux with detected anomalies using STL + Z-score, indicating notable space weather events.

Figure 7(a) Actual vs. predicted satellite NN1_Voltage time series using the Random Forest model. Blue represents the observed values, orange denotes model predictions, and red markers highlight detected anomalies where the residual (actual – predicted) exceeds ± 1.5 standard deviations (σ). These NN1_Voltage anomalies are part of a broader set of **257 events** identified across all telemetry parameters using the same 1.5σ threshold. This set forms the basis for comparison with anomalies in space weather parameters detected using Z-score and STL decomposition methods.

For GCR (Fig. 7(b)), anomalies were distributed throughout the 2007–2010 period, with clustering during late 2008 and 2010. These periods correspond to the deep solar minimum of Solar Cycle 23, when GCR intensities peaked due to reduced heliospheric shielding. The anomalies reflect sudden decreases in GCR flux, known as Forbush decreases (FDs), which are often linked to interplanetary coronal mass ejections (ICMEs). Although FDs represent decreases in GCR intensity, they mark intervals of highly disturbed interplanetary conditions that can degrade satellite electronics and power subsystems through associated SEP events and geomagnetic storms.

For P10 proton flux (Fig. 7(c)), anomalies were less frequent during 2007–2009 but increased sharply in 2010, with more than 40 events detected. These enhancements coincide with the onset of Solar Cycle 24, when solar energetic particle activity intensified. P10 anomalies are particularly critical because energetic protons deposit ionization and displacement damage in solar arrays and electronics, directly contributing to satellite performance degradation.

Although direct fault confirmation was not possible due to the lack of engineering logs, the statistical consistency between detected anomalies and external space-weather stressors supports their interpretation as genuine subsystem irregularities rather than model artefacts.

**Fig. 8:**
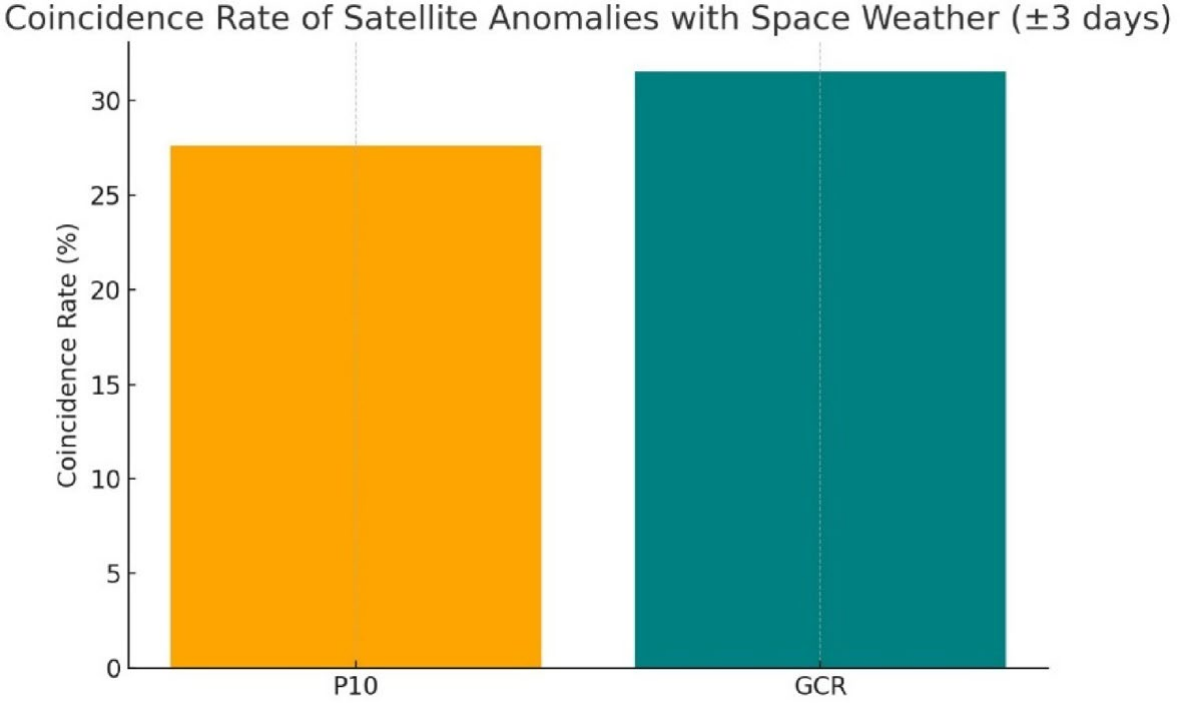
Coincidence rate (%) of satellite anomalies with space weather parameters (± 3-day window). The orange bar represents anomalies coinciding with high-energy proton flux (P10), while the teal bar corresponds to anomalies coinciding with Galactic Cosmic Ray (GCR).

Table 7 present the coincidence rates between satellite anomalies and space weather events (GCR flux variations and P10 proton enhancements) within ± 1, ±3, and ± 5 day windows to assess sensitivity. For each window, we computed observed coincidence rates between satellite anomalies and space weather disturbances (GCR and P10) and compared them against permutation-based null baselines with 95% confidence intervals. As in Table 7 at ± 3 days window, 31.5% of anomalies coincided with GCR events compared with a null expectation of 56.5% (95% CI: 48.2–65.4%), yielding a statistically significant deviation (χ² = 60.6, *p* < 10⁻¹⁴). Similarly, P10 coincidences at ± 3 days were 27.6% versus a null expectation of 45.1% (95% CI: 37.0–52.9%; χ² = 67.3, *p* < 10⁻¹⁵). The negative effect sizes reported in Table 7 arise because the null mean coincidence rate (pₙ_u_ₗₗ) is higher than the observed rate (pₒ_bₛ). This occurs when real space-weather anomalies (GCR or P10) cluster in time—often around specific solar-cycle phases such as solar minimum, whereas the random permutations used to generate the null distribution spread events more uniformly throughout the timeline. This uniform redistribution increases the probability that some random space-weather dates fall within ± N days of satellite anomalies, resulting in higher pₙ_u_ₗₗ and hence negative Δp values. Despite this negative direction, the χ² and Fisher tests show very low p-values, confirming that the timing of anomalies departs significantly from randomness. Physically, this pattern indicates a lagged or cumulative response, where satellite anomalies are more likely to occur during recovery or Forbush-decrease periods rather than exactly at the GCR or proton-flux peaks. We also emphasize that the ± 3-day window is used as our main analysis window in line with previous studies, which report that the effects of both galactic cosmic rays and solar proton events on satellite subsystems typically manifest within 1–3 days of the disturbance^49^. This choice ensures physical consistency between space weather disturbances and their expected timescale of impact on spacecraft telemetry. Figure 8 illustrated ± 3 days is selected as the primary analysis window as it reflects the typical response timeframe of spacecraft systems to space weather disturbances.

**Table 7:** Statistical association between satellite anomalies and space weather events for (± 1, ± 3, and ± 5 -day window), including Chi-square and fisher’s exact test values.

Metric	Window	Observed Count	Observed Rate	Null Mean Rate	95% CI Low	95% CI High	Effect Size	Chi2	Chi2 *p*	Fisher *p*
**GCR**	± 1d	42	0.163	0.3	0.245	0.358	−0.137	4.73	2.97e-02	3.98e-02
	± 3d	81	0.315	0.565	0.482	0.654	−0.25	60.64	6.85e-15	2.64e-13
	± 5d	113	0.44	0.729	0.638	0.809	−0.289	138.54	5.55e-32	3.73e-28
**P10**	± 1d	44	0.171	0.226	0.179	0.28	−0.055	17.08	3.57e-05	9.07e-05
	± 3d	71	0.276	0.451	0.37	0.529	−0.174	67.31	2.33e-16	2.86e-14
	± 5d	90	0.35	0.611	0.506	0.696	−0.26	115.3	6.78e-27	3.31e-23

## Conclusion

This study presented a four-stage machine-learning framework for investigating how space-weather dynamics influence satellite power performance, using EgyptSat-1 as a case study. By integrating advanced feature selection, multivariate regression, and anomaly validation, the framework offers a quantitative, data-driven approach to examine the nonlinear coupling between solar-terrestrial conditions and spacecraft telemetry. The two-stage feature selection identifies the most influential drivers, namely: galactic cosmic ray (GCR) intensity and the ≥ 10 MeV proton flux (P10), while maintaining physical interpretability and robustness across conditions.

Among the six predictive models assessed, Random Forest achieved the best overall accuracy for both NN1_Voltage and TBS1_Current, with mean absolute percentage errors of 0.09% and 2.4%, respectively. Lag-depth validation indicated that a three-day temporal memory best captured the delayed response of the satellite power subsystem to cumulative space-weather forcing, consistent with previously reported 1–3-day coupling timescales between solar-energetic-particle activity and satellite anomalies. The anomaly-detection stage, combining STL decomposition and Z-score analysis, successfully isolated both transient and periodic variations in GCR and proton fluxes. Statistical comparison revealed significant temporal coincidence rates 31% for GCR and 27% for P10 within ± 3-day window validated through chi-square and Fisher’s exact tests (*p* < 10⁻³). These results suggest a measurable link between radiation-driven disturbances and short-term fluctuations in EgyptSat-1’s power telemetry, providing new quantitative evidence for space-weather-induced degradation mechanisms.

The framework’s applicability is currently limited using data from a single satellite and mission phase. Extending the analysis to additional satellites and incorporating broader environmental and operational variables would improve generalization. Future work will focus on validating the detected anomalies using missions with verified engineering logs and explore hybrid physics-informed and data-driven approaches to enhance interpretability and robustness.

Overall, the results indicate that machine-learning-based analysis can support a deeper understanding of satellite–environment interactions and contribute to developing more resilient power-system diagnostics and health-monitoring strategies.

## Data Availability

All reproducibility artifacts, including the cleaned space weather dataset, runnable Jupyter notebooks, and a README with instructions, are available at [https://github.com/Hamada224/EgyptSat-1-reproducibility.git](https:/github.com/Hamada224/EgyptSat-1-reproducibility.git).
